# Ethics review, reflective equilibrium and reflexivity

**DOI:** 10.1177/09697330211003252

**Published:** 2021-07-28

**Authors:** Julie Morton

**Affiliations:** University of Salford, UK

**Keywords:** Ethics review, reflective equilibrium, reflexivity, research ethics, research ethics committees

## Abstract

**Background:**

Research Ethics Committees (RECs) or their equivalent review applications for prospective research with human participants. Reviewers use universally agreed principles^i^ to make decisions about whether prospective health and social care research is ethical. Close attention to understanding how reviewers go about their decision-making work and consider principles in practice is limited.

**Objective:**

The study aimed to understand how reviewers made decisions in the contexts of meetings and to understand more about how reviewers approach their work. The purpose of this article is to draw on data and findings and to show how reflective equilibrium as a theoretical frame can (1) deepen understanding of ethics review and (2) permit a reflexive examination of the habitual processes of review.

**Design and participants:**

Methods captured the day-to-day work of the RECs. Seventeen applications were heard during eight observations. There were 12 formal interviews with reviewers (n = 12) and with researchers (n = 8) which are not reported on in this article.

**Ethical considerations:**

Organisational permission for the study was given by the National Research Ethics Service (NRES) whose functions became part of the Health Research Authority (HRA) during the study. The study was given favourable opinion by the University of Salford's REC (Reference HSCR11/17).

**Findings:**

Data were analysed using constructed grounded theory resulting in eight themes which revealed attention to procedure and engagement with applications. Reflective equilibrium was used as a qualitative frame to interpret themes distilling them into three processes at work in review: emotion and intuition; imagination and creative thinking; and intuition and trust.

**Discussion:**

Reviewers went back and forth between universal principles and considered these in the contexts of each application using the above processes.

**Conclusions:**

Reflective equilibrium offers a coherent and grounded account of review work. Reflexivity in training for reviewers is essential for improving practices. The challenges reflexivity presents can be assisted by using reflective equilibrium as a tool to illuminate tacit review processes.

## Introduction

National Health Service (NHS) Research Ethics Committees (RECs) in England are authoritative bodies. They function as the institutional mechanism through which society exercises jurisdiction over what kinds of research are ethical. This article draws on empirical research carried out with RECs. It uses examples from observations and interviews to show how reviewers apply principles in practice and tacitly use reflective equilibrium as a way of arriving at balanced decisions. Reflective equilibrium as method can be used as a means of reflective consideration of ethical and moral questions and has previously been proposed as a way of enhancing professionals’ ethical education.^1^ Reviewers use reflective equilibrium implicitly. None of the reviewers interviewed described their work in these terms; however, analysis of data derived from observations and interviews showed that reviewers do use reflective equilibrium in the practice of ethics review and it closely describes their work. In this article, the claim is that reflective equilibrium is tacitly used by reviewers, it describes the work of ethics review and a reflective equilibrium frame may helpfully be utilised for reviewers to understand better how they reach decisions. That is, a reflective equilibrium frame has the potential to describe, illuminate and enhance reviewers’ reflexive capacity to enable them to access the tacit processes they use in reaching decisions.

Ethical principles are formalised and codified through highly developed procedural systems. RECs or their equivalent (e.g. institutional review boards (IRBs) in the United States) review applications for prospective research to assess whether the proposed research is ethical. In the United Kingdom, RECs function as the committees where decisions about the *suitability* of research are made. Given their significant regulatory function, critique has been inevitable, though close attention to understanding how reviewers go about their work is limited. Reviewers on committees decide on suitability in a formal sense through checking whether applications and researchers comply with universally agreed principles which are operationalised in the form of digital applications in required consent forms, participant information sheets and in general statements about ethical conduct in research. In practice, reviewers used a range of tacit strategies to reach decisions. Tacit knowledge describes hidden assumptions and meanings transcending the immediate surface and yet guiding actions whether individuals explicitly say so or not.^2^ Reviewers balanced principles and tacit processes to reach decisions.

Rawls^3^ in a Theory of Justice described reflective equilibrium as a method of bringing into balance considered principles, judgements and theories into a state of harmony. This is ‘reached after a person has weighed various proposed conceptions’ (p. 43).^4^ Rawls developed and refined the meaning of reflective equilibrium but the term has wide application and is used in bioethics and clinical ethics broadly as a way of thinking through moral questions, a method of reflection for moral problems.^5^ ‘Wide’ reflective equilibrium (which Rawls referred to following his original discussion) incorporates a ‘wide’ scope of judgements, principles, rules, moral beliefs and intuitions to reach a balanced assessment of a problem. Reflective equilibrium has been recognised as a useful concept for bioethics because accounts from ‘the top’ (principles and rules) and ‘the bottom’ (cases and judgements) both need supplementation.^6^ This holds resonance for ethics review where decisions are reached by reviewers going back and forth between rules (principles and procedure) and particular cases (applications for review). The lens of reflective equilibrium was utilised in the analysis presented here as a descriptive frame which provided a credible explanation of what was happening in review work. This retrospective analysis is useful on two counts. First, it describes closely the effort and work which goes into reviewers’ deliberations. Second, the description of REC work as reflective equilibrium offers potential for learning and improving ethics review.

### The reach of ethics committees

There is no escaping ethical review by an appropriate committee for most research conducted with human participants. This includes virtually all research conducted in the United Kingdom, North America and European countries.^7^ There is global concern with ethical conduct in research. The World Health Organisation (WHO) proposes that all research with human beings should be reviewed by an ethics committee to ensure ethical standards are maintained and there is published guidance for the establishment and conduct of such committees.^8^ The Belmont Report (1979) recognised the complexity of ethical situations and the difficulties in interpreting rules embedding a broader principles approach as a basis for rules to be devised, criticised and interpreted.^9^ The foundations for ethical conduct in clinical practice and research are underpinned by the deontological approach usually summarised in the four principles of beneficence, non-maleficence, autonomy and justice.^6,10^ Principles are formalised and codified through highly developed procedural systems with RECs or their equivalent (e.g. IRBs in the United States) acting for larger institutions as arbiters of what constitutes ethical research. In the United Kingdom, the Health Research Authority (HRA) established in December 2011 aimed to promote and protect the interests of patients in health research and streamline the regulation of research. The most recent iteration of principles can be found in the framework for health and social care research.^11^ This far-reaching document incorporates core principles but in all outlines 19 points which act as a benchmark that research is expected to meet. The HRA’s brief includes responsibility for overseeing research in health and social care. RECs are charged with the review of applications for research which involve human research participants. Their work is broadly summarised as reviewing applications for research and providing ‘opinions’ about the proposed participant involvement and whether the research is ethical. Researchers in the United Kingdom are encouraged to attend REC meetings. The outcome decisions available to each REC are categorised as opinions and are currently ‘Favourable opinion’ (usually with conditions); ‘Unfavourable opinion’; ‘Provisional opinion’ (with request for further information); ‘Provisional opinion’ (pending consultation with a referee); or ‘no opinion’.^12^

### Criticisms and current debate about ethics review

RECs can in effect veto research with consequences for the creation of knowledge to support improvement and development of practice in health and social care fields. Formal review has therefore unsurprisingly been subject to extensive critique. Criticisms of formal ethics review (and oversight committees generally) are with the idiosyncratic nature of decision-making^13^ and concern with the role of ethical principles in ethics review which are utilised as ‘prescriptions’ for, and ‘proscriptions’ required of, researchers.^14^ Dissatisfaction with committees’ decision-making cannot simply be explained away as a by-product of researchers’ disappointing personal experiences. However, reviewers occupy a liminal space. In reaching a decision, they are considering both researchers’ desire to conduct research to produce knowledge and the rights of the researched to be protected in that process. Decisions are made by and between reviewers in the context of committee meetings. Thus charges of idiosyncratic decision-making and the use of principles to delimit research need to be based on what happens at meetings and what reviewers say about their work not just the resulting decisions.

Two types of criticism have been delineated by Sheehan.^15^ First, criticism of the shortcomings of the governance system and how it functions (these can be summarised as over-bureaucratisation, inconsistency, relevance for qualitative research and ineffectiveness) and the second, a theoretical critique which questions the need for RECs at all. Sheehan goes on to argue that critique requires evidence which is not always apparent in the arguments for change. Furthermore, attention would first need to be paid to whether the current system can evolve and develop in response to any existing problems. Apart from a few notable exceptions,^16–21^ both criticisms and suggestions for enhancement or development of RECs are not grounded in the work that is carried out. Limited attention is paid to the ways in which review work could improve, though there has been some recent debate about how committees should develop. Such debate has argued either that RECs should focus on consistency in the use of formal codes^22^ or whether reviewers’ ethical reflection and deliberation should be extended and improved.^23^ Other commentators have called for a professionalisation of RECs precisely because their role goes beyond checking for adherence to codes but importantly involves making judgements about the science, methodology and even research teams when they consider applications.^24^

While this critical commentary has spotlighted some of the challenges for the national framework of ethics in health research and raises important issues that require redress, ideas and recommendations are predominantly not grounded in empirical data. In other words, there is a limited evidence base in terms of what reviewers do in meetings, on *how* work is accomplished, and decisions made. Critique does not generally engage with the language and concepts used by reviewers^25^ including how they use gut feelings. Even in studies utilising established quantitative frameworks to ‘measure’ performance and adherence to procedure there is acknowledgement of the scant data on the working of RECs globally, that is, ‘what they do and why they do what they do’.^26^ A focus on work – in the settings of meetings and what reviewers say they do – is largely absent in extant literature. Furthermore, frames for describing and interpreting the work are needed. Here, reflective equilibrium offers a helpful way of both describing and theorising about review work and, a potential way in which reviewers could examine what they do and how they do it thereby developing their reflective ability.

### Reflective equilibrium in review work

Rawls described the two elements of the term reflective equilibrium: ‘Equilibrium’ because principles and judgements coincide and ‘reflective’ because ‘we know to what principles our judgements conform…’ (p. 18).^3^ For Rawls, reflective equilibrium as a method involves a back and forth activity where we constantly adjust judgements, matching these to ‘cases’, pruning judgements so that these along with other beliefs enable us to reach a decision that is coherent. As a method, reflective equilibrium is a reflective means of adjusting broad principles to render them coherent in the context of cases. Translating this for review work, reflective equilibrium is the consideration of how overarching principles need to be altered and applied to proposed research and in relation to other overarching principles.

Reflective equilibrium has flaws as a method. It describes how judgements are based on intuitions and what we feel is morally acceptable. Such judgements are contextualised, shaped by social and cultural norms. Nevertheless, reflective equilibrium is used here to reveal the dynamic nature of review work and how crucial decisions are made in RECs. Rawls acknowledged that reflective equilibrium as a method was not to discover moral ‘truth’ but was a descriptive project which enabled a better understanding of the moral sensibilities we have.^27^ The reflective equilibrium frame illuminates the processes of decision-making in RECs and as it is a reflective method, holds potential for revealing some of the tacit processes used by reviewers to reviewers themselves.

## Method

### Design

The data reported on in this article were collected during the course of an institutional ethnographic study using methods which captured the day-to-day work of the REC as sites where review *happens*, and decisions are made about whether research is ethical. Implicit in the focus on day-to-day work of organisations and the ‘mundane’ is recognition that knowledge can be generated about institutions and the ways in which their work is tied together and co-ordinated. This can lead to findings of wider social significance as well as illuminating practices for people working within them.^28,29^

### Setting and participants

NHS RECs consist of up to 18 members, one third of whom are lay (broadly, this means their main professional interest is not in a research area, nor are they a registered healthcare professional). Their work is voluntary. Reviewers began meetings by having a general discussion about the application under review led by two reviewers who had been tasked with a close reading. The Chair collated questions and queries for the researcher who would come in following the preliminary discussion. Following the discussion with the researcher, an opinion (decision) was reached through continued deliberation.

### Ethical considerations

Organisational approval for the study was given by the National Research Ethics Service (NRES) whose functions became part of the HRA during the study. They had oversight of the study design and advised on consent arrangements. The study was given favourable opinion by the University of Salford’s REC (Reference HSCR11/17). Consent was initially negotiated directly with the Chairs of RECs who sought individual (reviewers) and committee consent for the research. This allowed reviewers to consider whether they wanted to be involved prior to formal consent being taken. Participant Information Sheets were provided to individual panel members at observation and interview stages. These explained that the purpose of the research was to investigate how reviewers debated and thought about applications and how they arrived at decisions about ethical research with vulnerable people. Information provided aligned to the methodological foundation of the study which was to explore how applications were conceptualised (thought about and discussed) in meetings and how review work was described by *those who were doing the work of review.*

Decisions were communicated to me via the national co-ordinator. Not all RECs consented. Individual consent was again sought for interviews.

### Data collection

Observations took place over a period of 18 months and typically lasted between 1 and 3 hours. Close field-notes were taken as audio recording was not permitted. Observations at meetings allowed access to the practical work of ethics review – paying attention to reviewers’ deliberations in situ. Follow-up interviews permitted insights into the reviewers’ own interpretation and understanding of what their work was about. Through observations and interviews with reviewers, data were gathered on the everyday of review work and reviewers’ perceptions of what they did.

Seventeen applications were heard during eight observations. There were 12 formal interviews with reviewers (n = 12) and with researchers (n = 8) (not reported on in this article).

### Data analysis

Descriptive codes were constructed using a grounded theory approach.^30^ Grounded theory views coding as ‘constructed’ because it reflects the views of the researcher, reflecting subjective choices about what is seen as significant and using the researcher’s choice of words (p115). To this extent, the analysis adopted a grounded theory approach.

Theorising from the data led to a description of work which encompassed objective and subjective processes. The use of institutional texts was a strong feature and is reported elsewhere.^31^ Having discovered that reflective equilibrium seemed a close approximation of what reviewers did in meetings and described their work, reflective equilibrium was used further as a qualitative post hoc frame of analysis, as a theoretical lens to tell the story of what happens in ethics review. It is not intended to provide a complete, definitive understanding of the work of RECs, however, reflective equilibrium illuminates processes at meetings potentially providing insights for reviewers in assisting a reflexive approach to their work as well as helping researchers to understand how reviewers work to reach decisions in a largely reflective manner, moving between regulations and intuitions to reach a reasonable judgement.

The descriptive codes and how they illustrate features of reflective equilibrium are shown in Table 1. A focus on the ‘bottom up’ features of reflective equilibrium resulted in the identification of three forms of tacit process.

**Table 1.** Themes and congruence with features of reflective equilibrium.

Themes extrapolated from data	Frequency across data sets	Features of reflective equilibrium in review work	Tacit processes used in reaching equilibrium between principles and moral reflection
Concern with design – seeking understanding	21	**‘Bottom’ – engaging with cases** Situated in context of application Particular Characterised by achieving the ‘right’ moral outcome Involves engagement with research and researcher using emotion, moral reflection, intuition and judgement	Emotion and intuition Imagination and creative thinking Intuition and trust
Concern with ‘good’ research – feeling it had to be worthwhile, imagining experience	31		
Judgements about researcher – trusting	19		
Moral reflections and emotion, imagining	32		
Concerns with language	7	**‘Top’ – principles and procedure** Universal Procedural Principle-led Rules Characterised by achieving the ‘correct’ procedural outcome. Involves administrative checking, correct paperwork and conforming to institutional process	
Referring: References to paperwork	24		
Referring: Specific reference to ethical requirements – autonomy, equality	9		
Deferring: Seeking advice/clarity on ethical requirements	19		

## Findings

The tacit ways reviewers reached decisions were by using ‘emotion and intuition’; ‘imagination and creative thinking’; and ‘intuition and trust’, balancing these with principles. In practice, they referred to universal principles and procedure, but they did more than simply check adherence to these. They also focussed on the sensitivities presented in each application. The data extracts illustrate this back and forth process. Reviewers are making crucial judgements about whether applications measure up to legal and procedural requirements and the ethical imperatives of autonomy, beneficence and justice. However, principles are only put to work through a range of other forms of engagement with the applications under review.

### Emotion and intuition

Themes of engaging with design, moral reflection and concern with good research were ways in which reviewers brought meaning to ethical principles in the context of the applications considered.

For Rawls, ‘considered judgements’ may be judgements which we all broadly agree on, such as protection of research participants from harm, but this might not be an absolute principle when we consider possible conflicts in the contexts of particular cases. The challenges are the tensions in what constitutes harm, and this differs according to context. This was illustrated by a reviewer reflecting on the meaning of harm in research using the example of blood-tests and who said:

If we’re taking blood samples from babies, then the baby screams – there is distress to the baby and the mother (sic) parent) so we have to question how many times we can take blood. What is reasonable? Clinicians (as researchers) may treat these situations in the same way even when in one case the blood tests might be for treatment and in another, for research. What if it’s not to do with treatment – it’s not clinical judgement but research judgement. The question of burden has to come up.

The challenge is the tension regarding what constitutes harm. The broad principle (which according to Rawls, our intuitions are based on) can be agreed, but the conceptualisation in the context of specific cases is difficult. How this is balanced against other principles (as in the next example) is a further thorny issue in ethics review.

Reviewers had to decide on what was reasonable harm or burden in an application considering a novel form of communication for people with dementia. The researcher was a postgraduate student. Following the observation, I discussed the decision with one of the lead reviewers. The committee had struggled with the principles of beneficence and non-maleficence because they felt it was unlikely that any startling advances could be made for this community of participants. The procedural requirements were a concern here because in the United Kingdom, the Mental Capacity Act 2005 (MCA 2005)^32^ requires that to involve participants who lack capacity to consent, research must have the potential to benefit the participant or be intended to provide knowledge of causes or treatment of the care of persons affected by a similar or the same condition.

Another reviewer said that s/he began with quality of research, ‘for example, are there well-defined objects, how will outcomes be managed. Most people are not evil who are doing research’.

And on vulnerability, ‘well there are certain categories like children or people with mental problems…As a community we have a responsibility not to authorize a study if it is only for the sake of achieving a higher degree. Research involves human beings. It sounds pompous, but it is our duty. People with dementia for example, we have to do our utmost to ensure that they are not used for something that is not worthwhile. Design is not our concern however bad research or bad science is not ethical’.

This balancing of harm and benefit, the weighing up of universally accepted principles where risk–benefit relations are conceived ‘in terms of a ratio between the probability and magnitude of an anticipated harm’ (p. 230)^6^ is an everyday part of review work. Reviewers do this using emotional engagement, thinking themselves into the research to decide. The reviewer concluded that the researcher wanted to get the best form of reaction they could when using this new form of communication, ‘....whether people were more settled, whether they smiled. People can be very isolated. There may be processes going on that we don't know. It's a great sadness’.

This kind of reflection drawing on feeling and emotion shaped review work. The specific role of emotion is not highly developed in reflective equilibrium but may be viewed as part of the intuitions and gut feelings that are deployed in reaching a balanced view of a problem. Emotions play a part in decision-making with emotions shaping reactions and ultimate decisions.

### Imagination and creative thinking

Another way in which reviewers engaged with design was through imagining the implications of research. Imagining themselves into the research was a way of resolving the inevitable tensions when applying principles. A reviewer who described her/himself as a lay member was reflecting on a study which had proposed to trial a new pharmaceutical regime for patients admitted to A & E, some of whom may have been unconscious and unable to consent.

The REC guidelines are difficult to keep in mind–I’m a lay member. I try to keep in mind what could go wrong for the patient, I try to be creative in my own mind and (I) asked the question what’s the worst that can happen? In this study if I was incapacitated would I want this to happen? And if I would want it, are there other reasons why someone would not. My gut reaction is – is this okay?

Gillam et al.^31^ used the term ‘imaginative identification’ to describe a common approach taken by reviewers where they imagine a close family member or themselves as participants in research under review. This is described as a non-abstract way of considering risks, disadvantages and possible benefits. It is another tacit technique used in reaching equilibrium between the reviewers’ concerns or unease about research and resolving the tension to be satisfied that ethical principles of harm and benefit are being met. Principle-informed procedures are amenable to a variety of interpretations. Codes are ‘inescapably normative concepts requiring normative knowledge, reflection and interpretation’.^23^ Using emotion to connect with the perspectives of participants and their carers is a way of making principles come to life.

An application which potentially included people who may lack capacity illustrated how reviewers engaged with the principle of fairness. The study proposed concerned the incidence of co-morbidities in patients admitted to hospital with heart conditions. The range of symptoms to be captured included mental and physical health conditions. Some participants were likely to have fluctuating capacity or permanent cognitive impairments. The concerns of the committee were with both the legal requirement in the MCA 2005 (S31-33)^32^ (that the study could not take place without the inclusion of those lacking capacity) and the principle of justice which can be understood here as ‘fairness’ or wanting to support the involvement of everybody in research. The discussion went as follows:

Reviewer 1: how would the study be affected if you didn’t include people who lacked capacity?

Researcher: Impairment is frequent in admissions and so this would cause the data to be biased. Some people admitted to the heart unit have dementia for example.

Reviewer 2: Well some patients might be confused but that would be temporary so you could go back to those patients. Can’t you exclude people with long term impairments?

Researcher: I don’t want to do this. Some people will have cognitive impairments.

Reviewer 3: (appealing to other reviewers) I struggle with this. If I compare this to my area, learning disability, I’d want to be encouraging about inclusion. People can communicate emotions such as pain and so on.

Researcher: I don’t want to exclude people on that basis. They may well be able to describe their symptoms, but they may not be able to consent to research. I could have excluded people (who can’t consent) but I think that would be the easy option.

When the researcher left, the discussion turned to shortcomings in relation to the consent documentation. The debate between reviewers balanced the ‘flawed’ application with the importance of including participants who could not consent in the research to pursue knowledge.

S/he wants to include (this group of people) in the pursuit of knowledge so why should we stand in her way?

It’s flawed but maybe it’s as good as it can be.

Here, to make a judgement, reviewers were faced with the problem of an application which had not fully addressed the principle of autonomy (operationalised as consent requirements) and so was procedurally inadequate. The reviewers took a view that people should be included in the research even if it is harder to negotiate their consent or legally seek consent by proxy (through a consultee). The discussion encompassed their moral sensitivities, intuitions and the squaring of these with the imperative for adherence to principle and procedure, specifically, principles of justice and autonomy. Reviewers’ deliberations operated at different levels of abstraction, some abstract, some concrete. Again, reflective equilibrium offers an insightful way of understanding these discussions as it describes a process which pays attention to moral and non-moral beliefs at different levels of reflection such as intuition, moral rules or principles and abstract theories.^5^

Resulting decisions in review are not objective but emerge from deliberations that incorporate individual judgements and gut feelings which give definition to overarching principles. Reviewers would be unable to arrive at a coherent position without finding an equilibrium between the abstract principles and their gut/intuitive reactions to the concrete case under review. Reviewers use creativity in thinking about cases. Discourses of objectivity and consistency may appeal but creative thinking, curiosity and reflection in the context of the research proposed are required to make decisions. The final example shows how trust and trustworthiness were also part of decision-making.

### Intuition and trust

Reviewers use what is available to them to make the best judgement calls on research. Procedure could be trumped for example by estimations of trust and trustworthiness. One reviewer talked about the role of trust in the researcher, how she or he made a judgement about trusting and how this was privileged over the ‘minutiae’ of procedure.

It’s not to do with their moral life but when they come in, what they show. Are they trustworthy, do they have integrity and an understanding of what they are doing? It’s kind of subjective – but not. (It’s) the way they answer questions, their conduct, their modesty, admitting/acknowledging mistakes…

So, (we’re) not bogged down with minutiae – (we are) willing to trust. It (the minutiae) becomes important if we’re not able to trust.

Stark^19^ described how reviewers in IRBs in the United States assessed researcher trustworthiness by reviewing the submitted documents. For example, researchers who had submitted a ‘tidy’ document using the correct language were more likely to be positively appraised. Estimations of trustworthiness are different in the United Kingdom because researchers are encouraged to attend meetings. Appraisal of the researcher is not solely based on the quality of submitted documents. Reviewers comment on the documents and whether consent forms comply with regulations and use the required language but they are prepared to step outside of that limited approach. Appraisal becomes another part of the deliberation using intuition and is based on the face-to-face interaction (Are researchers modest? Do they acknowledge mistakes?) with the researcher. Thus, the researcher’s attendance and resulting researcher/reviewer exchange provides an additional layer of information (besides the application) on which reviewers can reflect. This becomes part of working up to a moral case for approving research so that the end decision, though usually contingent, has been reached through a process of balancing procedure (the ‘minutiae’) with feelings and intuitions about the application and researcher.

Procedures operationalise principles and distill them into the required forms. Reviewers directly pursued concerns about omissions in the application. One reviewer talked about how it was possible to ‘lead’ the researcher into providing the ‘correct’ responses or responses which would lead to a favourable opinion. She or he described how

The onus is on the researcher to make the case, but I don’t know if you noticed but we fed her (the researcher) the lines. We have to draw out the bits to satisfy the legal requirements. We’re teasing bits out to satisfy ourselves. It makes it easier. The hurdles are low. If you use the (correct) language, you can pass

This reviewer’s comments seem to suggest that both parties (reviewers and researchers) engage in a kind of regulatory dance. Ethical principles are not fixed but abstract and open to interpretation. Procedure and regulation are institutional ways of ‘fixing’ principles. Nevertheless, principles require interpretation in particular contexts. In review, this interpretation of principles happens in the debate about the application, the description of prospective research and with the researcher. Where reviewers decided that research adhered (broadly) to principles but not to the letter of the regulatory requirements, they sought out the required language. Ultimately, procedure must be adhered to and decision letters sent. Just as the REC post-meeting letters to researchers are the result of a need for a single authoritative ruling on the ethics of an application^33^, the meeting itself has to conclude with a definitive and recognisable decision which can be translated into a script of requirements. These concern the ‘correct language’ and ‘the correct lines’ which are needed from the researcher so that formal review can be seen to have taken place.

To conclude, findings have shown that reviewers balance their responses to applications (intuition, emotion and imagination) with overarching principles and procedure in their deliberations, moving between the universal and the particular to reach a decision in each case.

## Discussion

The practical imperative of reaching a decision in ethics review can be explained using reflective equilibrium as a conceptual frame. This approach has illuminated how principles and procedure are apparent in review work but that these constantly interact with reviewers’ own ways of making sense of research. In wide reflective equilibrium, coherence or holism is achieved through reasoning ‘back and forth between judgments about cases, moral principles, and non-moral background theories, without giving epistemic priority to any of these elements of the method’.^34^ The back and forth in ethics review is between instinct, personal intuition about researchers, empathy, emotional engagement and imagination. Decisions are the outcome of consideration of a range of moral and non-moral feelings. Principle-informed procedure has an important role, in that review work uses procedural texts to make decisions appear rational and neutral.^35^ Nevertheless, the practical, emotional and subjective factors involved in decision-making go beyond the strictures of formal procedure. Much of review work is about finding coherence between these disparate elements, and finding a warranted solution to a practical moral problem is reached by questioning ‘the tenability and relevance of all sorts of beliefs, none of which is immune to revision’.^5^ Nevertheless, these processes are tacit. They are not obvious or amenable to scrutiny. They are the practical ways decisions are made but are not *available* for reflexive learning.

### The potential of reflective equilibrium to assist improvement of review work

Reflective equilibrium provides a frame which illuminates how reviewers reach a balanced judgement. This includes weighing up principles and their application – what they mean in the context of ‘cases’ (research applications). For Rawls, principles and cases have a reciprocal relationship, in that principles provide guidance while cases provide an opportunity for application and considered judgements, refining principles and making them relevant to individual, complex cases. In ethics review, principles inform and guide procedures. Reviewers must balance overarching principles of autonomy, justice, harm and benefit ratios with research applications which require a decision. Ultimately, reviewers are not simply involved in matching applications with procedure but use their own subjective judgements balancing these with what is permissible. The pace of meetings and the practical need for decision-making means there is little space for reflection. However, outside of meetings in interviews, reviewers demonstrated the ability to reflect on what they did.

Procedures in digital and paper form are imbued with ethical principles. Reviewers are tasked with applying abstract concepts which are only generally defined in regulations and other foundational documents.^36^ For Savulescu, subjective interpretation and context mean that absolute consistency is impossible to achieve. In response to the question of what RECs should do, he states, ‘The best answer I have found is reflective equilibrium, using their code, other international statutes, law, declarations, moral principles, theories and intuitions’.^23^ This article goes some way to providing evidence that reflective equilibrium does in fact closely describe the decision-making process. Reflective equilibrium (in this article) is suggested as a frame to (1) show that this closely describes what reviewers do and (2) proposed as a helpful frame to illuminate work for reviewers carrying the potential for improving and developing review work.

Moral deliberation of the kind that happens in RECs is a crucial part of decision-making. Research on observations of RECs in France, Germany and the United Kingdom highlighted that committee discussions were grounded in the particularities of the case considering design, quality of information and relevance of the study rather than using normative principles. This included reviewers bringing their own experience and backgrounds to bear on discussions.^21^ The use of such tacit means of decision-making needs to be examined so that processes can be scrutinised, and reviewers can access their established practices and reflect on them. One of the criticisms of reflective equilibrium as a method is that it seemingly affords credibility to intuitions (and other tacit responses or reactions to ethical questions). This acceptance can undermine the credibility of any debate (or subsequent decisions) because existing prejudices may be reinforced or reproduce moral conservatism.^1^ Intuitive reasoning is unavoidable but needs to be examined precisely because of these hazards. The reflective equilibrium frame presents opportunities for reviewers to examine the tacit, unconscious, intuitive, emotional and imaginative aspects of their practice which they use to reconcile the prescribed principle and procedural direction of review work. Ethics review is saturated with procedure and bureaucratic requirements. However, RECs could not accomplish their regulatory function if review work consisted solely of reference to abstract, principle-informed requirements and evaluating adherence to procedure. In fact, reviewers engage in deliberations which are often only tangentially connected to abstracted procedures. Decision-making was situated, concrete (in the here and now and familiar) and practical (making sense, as far as possible, of prospective research).

The strength of the study reported on in this article is that it starts with review work, engaging reviewers and what they say about their work as well as using observations of meetings. It reveals how reflective equilibrium closely describes reviewers’ work and explains how reflective equilibrium as a conceptual frame can be used to provoke a reflexive approach to development of RECs through training. There are limitations in this analysis. The perspectives of participants in research are not addressed directly and are obscured here as they generally are in the critiques of ethics review. Participants are rendered passive, remote from decision-making and denied agency. A serious consequence of institutionalised ethics review with its inherent authority and power is that ‘vulnerable’ research participants are rendered more vulnerable because of their construction as passive players and the potential for researchers to avoid research with such groups because of the bureaucratic hurdles.^37^ This should be a concern for all of us as potential researchers and participants in research. However, this article has contributed to the ongoing debate about RECs. It has shown how reflective equilibrium might extend our knowledge of RECs as working authoritative bodies. The illumination of *processes* and actual review work is particularly important in bureaucracies which generally privilege audit (outcomes) over process.

### The connection between reflective equilibrium and reflexivity

Reflective equilibrium as a method has wide application and is used in bioethics and clinical ethics broadly as a way of thinking through questions – a method of reflection for moral problems.^5^ Reflective equilibrium’s foundational endeavour is justice, through examination of intentions, obligations and moral duties. These intentions, obligations and feelings of duty are apparent in reviewers’ work and are used tacitly in reaching decisions. Reviewers’ training could be enhanced by examining what motivates their decision-making including scrutinising the intuitions, emotions and imaginative processes at work, then examining how these fit with intentions, moral duties and obligations. Balancing of obligation and feeling characterises reflective equilibrium as a method. The claim here is that reflective equilibrium closely describes what *actually happens* and can provide a useful tool for training. Reflexive analysis is a way of examining and evaluating how this ‘to and fro’ activity works in decision-making processes.

### Training for the practical improvement of ethics review

Current debates dichotomise the purpose and practice of formal ethics review into what they should do: adhere to codes or reflectively deliberate suggesting professionalisation because of the knowledge and skill required.^22–24^ Findings illustrate that reviewers already possess knowledge and skills as well as reflective abilities. What is significant for the development and improvement of ethics review is that those involved have the reflexive space to consider their work. However, reflexivity is challenging in practice. Practical reflexivity in organisations means to both *examine* habitual ways of seeing the worlds and thought and behaviour acquired from authoritative sources and *evaluate* ‘consolidated habits of perceiving, thinking, remembering, resolving problems and feeling’.^38^ The findings in this study revealed a range of responses and tacit strategies in review. Introducing reflexive approaches in training could help in evaluating and reviewing existing practices, but to *enhance* practices, reviewers would first have to access what they do in the world of everyday work. This is demanding and difficult because processes are so embedded. Reflective equilibrium could be used as a tool to assist with such a process as it helps to describe and ‘frame’ what reviewers do when making decisions.

## Conclusion

Much empirical research on ethics review is retrospective, in that it examines decisions resulting from review rather than exploring review practice itself. The research findings reported on here use data derived from the work of review (observations) and interviews (with reviewers about their work). RECs’ *performance* would benefit from a more reflexive approach in training instead of a focus on legal and procedural requirements. Regulatory review systems are well established and unlikely to disappear, though they can evolve and change. Support and training offered seems to offer few opportunities for REC communities to reflect on their work. Whether reviewing prospective research in health or social care, a reflexive approach to decision-making is required. Reviewers do not need training to become more familiar with procedures. Meaningful development must start from the work itself with a reflexive examination of how decisions are made. Reviewers tacitly use reflective equilibrium as a method in reaching decisions. If (as has been argued) their reflective expertise is to be improved when making normative or ethical judgements,^23^ then the nature of training itself is important.

Reflexivity in learning is challenging because it requires an unearthing of what is done in the everyday to accomplish tasks, and this is tacit and not brought out into the open. Reflective equilibrium has been used here as a frame to interpret work processes. It can also potentially be used as a conceptual tool for reviewers to consider *how* decisions are made. This kind of reflexive analysis of review work is needed but alongside this, the nature of training is important if ethics review work is to evolve and improve. Practical reflexive approaches view learning as embodied and existential considering both how we feel and how we respond to others.^38^ Enhanced training would therefore involve researchers and participants as well as reviewers in learning and research communities.

## Note

i
https://www.wma.net/policies-post/wma-declaration-of-helsinki-ethical-principles-for-medical-research-involving-human-subjects/
